# Kidney-Tonifying Recipe Can Repair Alterations in Adrenal Medullary Chromaffin Cells in Asthmatic Rats

**DOI:** 10.1155/2012/542621

**Published:** 2012-03-11

**Authors:** Cheng-Ping Hu, Jun-Tao Zou, Ye-Qiang Zou, Xiao-Zhao Li, Jun-Tao Feng

**Affiliations:** ^1^Department of Respiratory Medicine, Xiangya Hospital, Central South University, Changsha 410008, China; ^2^Department of Respiratory Medicine, The First People's Hospital of Changde, Changde 415003, China

## Abstract

Traditional Chinese medicine suggests that renal deficiency is a causative factor of asthma, and tonifying kidney drugs are believed to be an appropriate and beneficial treatment. The adrenal medullary chromaffin cells (AMCC) transition to the neuronal phenotype is known to occur in asthma, as evidenced by degranulation of chromaffin granules, decline of epinephrine (EPI) and phenylethanolamine-n-methyl transferase (PNMT), and obvious alterations in cellular architecture. In this study, rats were sensitized and challenged with ovalbumin, then treated with Kidney-Tonifying Recipe (KTR) to evaluate the therapeutic effect. Tissues were evaluated for changes in pathology and EPI, PNMT, and peripherin expression. Degranulation of chromaffin granules and appearance of neurite-like process were found in AMCC from asthmatic rats, and these changes were corrected by KTR treatment. EPI and PNMT expressions were decreased in asthmatic rats and increased by KTR treatment. Peripherin expression was increased in asthmatic rats and decreased in the KTR-treated group. Morphological changes and decreases in EPI were observed when cultured AMCC were exposed to sera from asthmatic rats *in vitro*, and these changes were attenuated with the addition of sera from KRT-treated rats. These results suggest that the Kidney-Tonifying Recipe is capable of repairing asthma-associated alterations in endocrine function and the ultrastructure of AMCC.

## 1. Introduction

Traditional Chinese medicine (TCM) considers renal deficiency as a principal cause of asthma. A tonification strategy for kidney is often used to treat asthma in TCM, and many consider this to be a highly effective therapeutic approach [1, 2]. Recent studies into renal deficiency have implicated an imbalance of the neuroendocrine system as an underlying cause [3–5].

Asthma attacks are induced by the dysfunction of cholinergic and adrenergic nerves and their cognate receptors. Since there is a lack of adrenergic nerves innervating the human airway smooth muscles, the relaxation of airways is mainly regulated by epinephrine (EPI) binding to adrenergic receptors. Adrenal medullary chromaffin cells (AMCC) are the primary cells that secrete EPI [6, 7], and are thus important elements of the neuroendocrine system. Asthmatic patients have been shown to have significantly decreased levels of endogenous EPI, suggesting that EPI release could be impaired in this disease process [8, 9]. Previous work by our group has demonstrated that EPI levels are decreased in asthmatic rats, as compared with control rats, and that this decrease may, at least partially, be mediated by a phenotype transition from AMCCs to neurons [10–12].

The role and mechanisms underlying the beneficial effects that are observed upon use of tonifying kidney drugs are unclear. We hypothesized that tonifying kidney drugs affect the structure and function of AMCC. In this study, asthmatic rats were treated with a classic TCM formula known as Kidney-Tonifying Recipe (KTR), and the effects of the kidney-tonifying drugs on the alteration of structure and function of AMCC in asthma were investigated.

## 2. Materials and Methods

### 2.1. The Kidney-Tonifying Recipe

KTR is composed of *Rehmannia glutinosa* (*Shudi*), oriental water plantain rhizome (*Zexie*), monkshood (*Fupian*), medicinal Indian mulberry root (*Bajitian*), Common Yam Rhizome (*Huaishan*), Indian Buead (*Fuling*), cortex cinnamomi (*Rougui*), salvia chinensis (*Danpi*), cistanche (*Roucongrong*), and *Cornus officinalis* (*Zaopi*). All of these natural drugs were provided by the Department of Pharmacy, Xiangya Hospital, Central South University. These drugs were mixed at a ratio of 10 : 10 : 3 : 10 : 10 : 10 : 10 : 10 : 10 : 6, and then boiled for 30 minutes three times in distilled water at a volume of 10-fold the dry weight of the herb mixture. The solution was heated until the drugs reached a concentration of 1.33 g/mL (crude drug/solution volume).

### 2.2. Experimental Animals and Treatment

All animals used in this study were conventionally bred 6- to 8-week-old male Sprague-Dawley rats (Experimental Animal Center of Central South University, Changsha, China). This study was carried out in strict accordance with the recommendations from the Guide for the Care and Use of Laboratory Animals published by the National Institutes of Health, USA. The protocol was approved by the Ethics Committee at the Asthma Research Institute, Hunan Province.

Twenty-four rats were divided into three groups using a random digits table (*n* = 8 per group): control rats (control), asthmatic rats (asthma), and asthmatic rats treated with KTR (KTR). The rats were treated as follows: on day 0 and day 7, the asthma and KTR groups were sensitized with an intraperitoneal injection of 100 mg of ovalbumin (OVA) (Sigma, USA), 200 mg of aluminum hydroxide (Sigma), and 6 × 10^9^ heat-killed *Bordetella pertussis* (Wuhan Institute of Biological Products, China) in 1 mL of sterile saline. The control rats were sham sensitized by using an intraperitoneal injection of sterile saline without OVA. The sensitized rats were exposed to 30 minutes of 1% OVA (wt/vol) aerosol every day from day 14 to day 28, while the control rats were exposed to aerosolized sterile saline only. KTR-treated rats received a gastric gavage with the KTR solution (10 g/kg) 40 minutes prior to exposure. Control and asthmatic rats received a gastric gavage with the same volume of distilled water into the stomach at 40 minutes before exposure.

### 2.3. Measurement of Bronchial Responsiveness

*In vivo *airway responsiveness to histamine was measured 24 hours after the last OVA challenge using whole-body plethysmography (PLY 3211, Buxco Electronics). Rats were treated for 2 minutes with aerosolized saline or increasing doses of histamine generated by an ultrasonic nebulizer, and airway resistance was measured. Histamine-induced bronchoconstriction was calculated as the index of percent increase in airway resistance as compared to the peak of the reaction with baseline airway resistance [11, 13].

### 2.4. Tissue Preparation

After determination of bronchial responsiveness, blood from each rat was obtained by cardiac puncture, and the serum was inactivated by heating at 56°C for 30 minutes, then filtered through a 0.22 *μ*m filter and stored in −20°C until use. The left adrenal glands were excised and fixed in 4% paraformaldehyde or in 2% glutaraldehyde stored at −80°C until use. After pulmonary artery perfusion, the right middle lung lobe from each rat was also fixed in 4% paraformaldehyde and stored at −80°C.

### 2.5. Medium Preparation

Medium A was prepared as a mixture of 85% DMEM serum-free medium (Gibco, USA) and 15% serum from experimental rats. According to the origin of serum, three Medium A subgroups were generated: serum from control rats (control serum), serum from asthmatic rats (asthma serum), and serum from KTR-treated rats (KTR serum). Medium B was prepared as a mixture of 15% fetal bovine serum (Gibco, USA) and 85% DMEM serum-free medium.

### 2.6. Primary Culture of Rat AMCC [14, 15]

Adrenal glands were obtained from conventionally bred 6- to 8-week-old male Sprague-Dawley rats. Medullae were carefully freed from the capsular and cortical tissue under sterile conditions and incubated with Hanks' balanced salt solution. Subsequently, medullae were dissociated with 0.1% type I collagenase dissolved in glucose-enriched phosphate-buffered saline for 40 minutes. After harvesting by centrifugation and resuspending in culture medium B, the cells were adjusted to a cell concentration of 5-6 × 10^6^/mL and incubated at 37°C. Medium was changed after 24 hours, and then every 48 hours thereafter, to remove dead cells and cell debris.

The cultures in medium B were maintained for a total of 5 days and divided into the three experimental groups for treatment with one of the different medium A subgroups described above. Seventy-two hours after the AMCC cells were treated with medium A (experimental serum), the cells and the supernatants were collected for subsequent analysis.

### 2.7. Hematoxylin and Eosin (HE) Staining

The tissue of lung and adrenal medulla were fixed in 4% paraformaldehyde and then embedded in paraffin. Tissue sections (4 *μ*m) were stained with HE, and the morphological changes of lung and adrenal medulla were observed under a light microscope. The airway wall thickness was quantified by pathology image analysis system.

### 2.8. Transmission Electron Microscopy

Adrenal medulla and AMCCs were fixed with 2% glutaraldehyde in 0.1 M cacodylate buffer, pH 7.2. Three hours later, specimens were postfixed in buffered 1% OsO_4_ for 1 hour following by hydrating in a decreasing ethanol series and embedding in Epon-Araldite. Ultrathin sections (70 nm) from the different specimens were stained with uranyl acetate and lead citrate and examined with an H-7500 transmission electron microscope (Hitachi, Japan). Ultrastructural changes were assessed by pathologists who were blinded with respect to treatments.

### 2.9. Enzyme-Linked Immunosorbent Assay (ELISA)

The levels of EPI, corticosterone, and NGF in serum or cellular supernatant were quantified by commercially available ELISA kit and antibodies, according to the protocol provided by the supplier (R&D, USA). Immunoreactivity was determined by an ELISA reader at 450 nm.

### 2.10. Reverse Transcription (RT) PCR

Total RNA was extracted from tissues or cells using Trizol reagent (Invitrogen, USA) according to the manufacturer's instructions. For RT-PCR reactions, primer sequences were designed that yielded 137 and 307 bp products of peripherin and GAPDH, respectively. The gene-specific primer sequences were as follows: 5′-GGTGGAGGTAGAGGCAACA-3′ (forward) and 5′-TCGGACAGGTCAGCGTATT-3′ (reverse) for peripherin, and 5′-TGAACGGGAAGCTCACTGG-3′ (forward) and 5′-TCCACCACCCTGTTGCTGTA-3′ (reverse) for GAPDH. The PCR amplification program consisted of preheating at 94°C for 5 minutes and 35 cycles of denaturing (94°C, 30 seconds), annealing (60°C, 30 seconds), and extension (72°C, 1 minute), followed by a final extension at 72°C for 10 minutes. The PCR products were electrophoresed on a 2% agarose gel to confirm size. Results were normalized to GAPDH, which was used as an internal control.

### 2.11. Immunohistochemistry

After embedding in paraffin at 4°C overnight, the tissues were sectioned (4 *μ*m) and mounted on slides. The tissue sections were then deparaffinized in toluene and rehydrated in a decreasing ethanol series. Endogenous peroxidase activity was blocked with 3% H_2_O_2_ for 10 minutes. The sections were then incubated with polyclonal rabbit antibodies against rat (anti-phenylethanolamine N-methyl transferase (PNMT), 1 : 2500; antiperipherin, 1 : 150; both from Millipore, USA) at 4°C overnight and followed by incubation with biotin-labeled goat anti-rabbit immunoglobulin (Ig)G (1 : 500; Zhongshan Goldenbridge Biotechnology, China) for 20 minutes. The sections were then treated with the streptavidin-peroxidase kit (Zhongshan Goldenbridge Biotechnology, China) and developed with DAB substrate. Counterstaining was performed with hematoxylin. Afterwards, the sections were incubated with nonimmune rabbit sera for negative controls. The results were observed under a light microscope (100x) and the average optical density was calculated by using the Image Pro Plus 6.0 software (IPP6.0) for subsequent statistical analysis.

### 2.12. Western Blot Analysis

Total proteins were extracted by RIPA Lysis Buffer (Takara, Japan). Thirty micrograms of protein were resolved by 10% sodium dodecyl sulfate-polyacrylamide gel electrophoresis (SDS-PAGE). Proteins were then electrotransferred to polyvinylidene fluoride (PVDF) membrane at 120 V for 1.5 hours. Nonspecific binding was blocked by incubating with 0.05 g/mL skim milk powder at room temperature (20°C) for 2 hours, and then membranes were incubated at 4°C overnight with rabbit anti-rat peripherin polyclonal antibodies (1 : 1000; Millipore, USA). After washing with PBST, the membranes were incubated at 37°C for 1 hour with secondary antibodies (1 : 5000; Sigma) and visualized by using the enhanced chemiluminescence reagent system (Pierce, USA). Results were normalized to GAPDH, which was used as an internal control.

### 2.13. Statistical Analysis

Data are presented as mean ± standard deviation $\left(\overset{̅}{x}\pm \text{SD}\right)$. One-way analysis of variance was used for multiple comparisons, followed by Fisher's protected least significant difference test. A *P* value of less than 0.05 was considered statistically significant.

## 3. Results

### 3.1. Airway Responsiveness to Histamine

**Figure 1:**
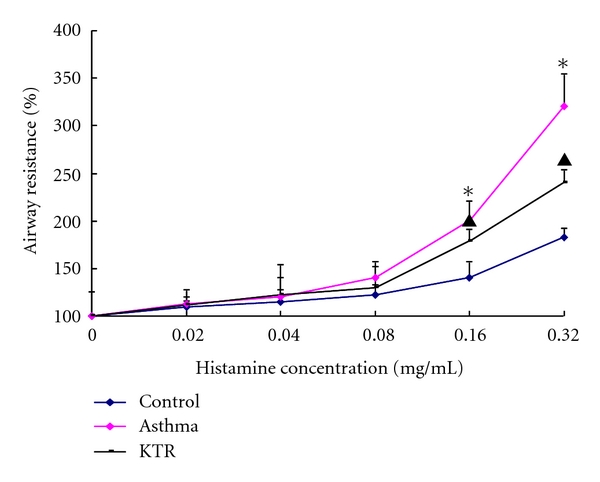
Changes in rat airway resistance in response to histamine exposure. Rats in three groups were treated with aerosolized saline or increasing doses of histamine generated by an ultrasonic nebulizer. Airway resistance was measured by whole-body plethysmography and expressed as the index of percent increase in airway resistance as compared to the peak of the reaction with baseline. The values are expressed means ± SD (*n* = 8). *indicates significant difference compared to the control group (*P* < 0.05); ^▲^indicates significant difference between KTR-treatment and asthma groups (*P* < 0.05).

Administration of increasing concentrations of histamine caused gradual increases in airway resistance in each group. When the concentration of histamine reached 0.16 mg/mL and above, airway resistance in the asthma group was higher than that in the control group (*P* < 0.05). KTR treatment attenuated the airway resistance in asthmatic rats (*P* < 0.05) (Figure 1).

### 3.2. Morphological Changes of Lung Tissue

**Figure 2:**
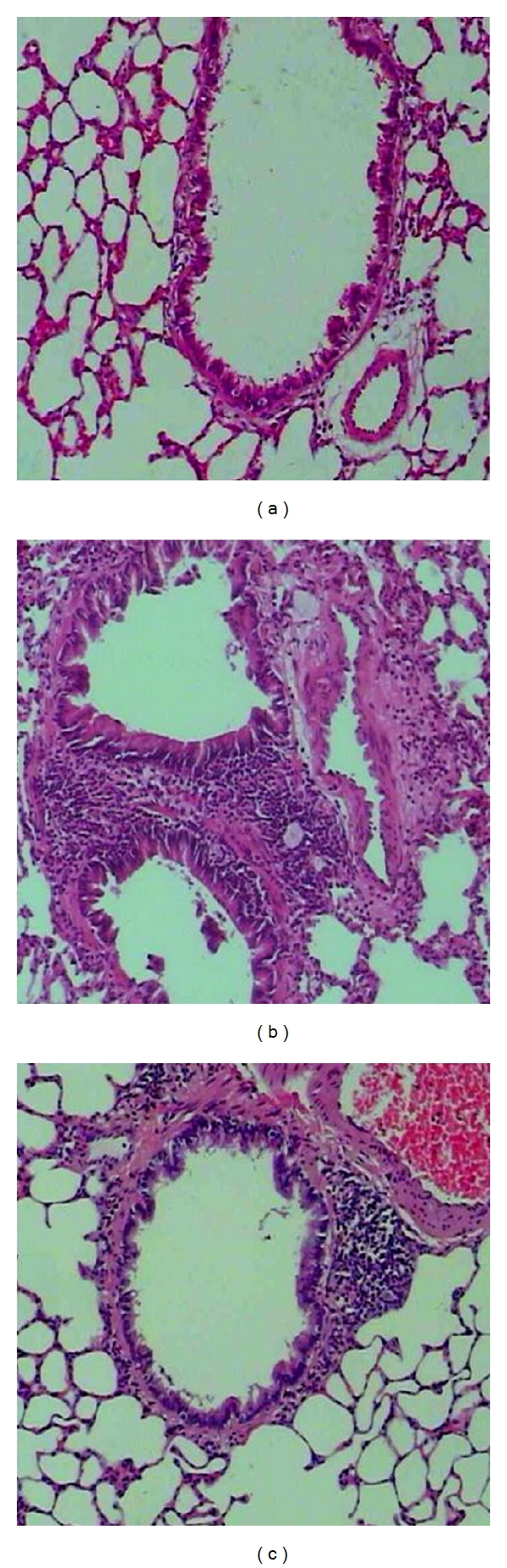
Pathological changes in rat lung tissue in response to induced asthma and KTR treatment. Representative micrographs (HE staining, 100x) of lungs from control rats (a), asthmatic rats (b), and KTR-treated asthmatic rats (c).

Morphological changes in lung tissue were evaluated by examining lesions in asthmatic rats. We found that, compared with the control rats, asthmatic rats had an increased shedding of epithelial cells, remarkable infiltration of eosinophils, and lymphocytes surrounding the airway. These pathological changes were found to be reduced in the KTR-treated rats. The results showed that the airway wall thickness in asthmatic rats (38.25 ± 4.98 *μ*m^2^/*μ*m) was thicker than that in control rats (18.88 ± 3.36 *μ*m^2^/*μ*m) (*P* < 0.05), and this effect was alleviated in KTR-treated rats (25.38 ± 5.42 *μ*m^2^/*μ*m; versus asthma, *P* < 0.05) (Figure 2).

### 3.3. Alterations in the Adrenal Medulla

**Figure 3:**
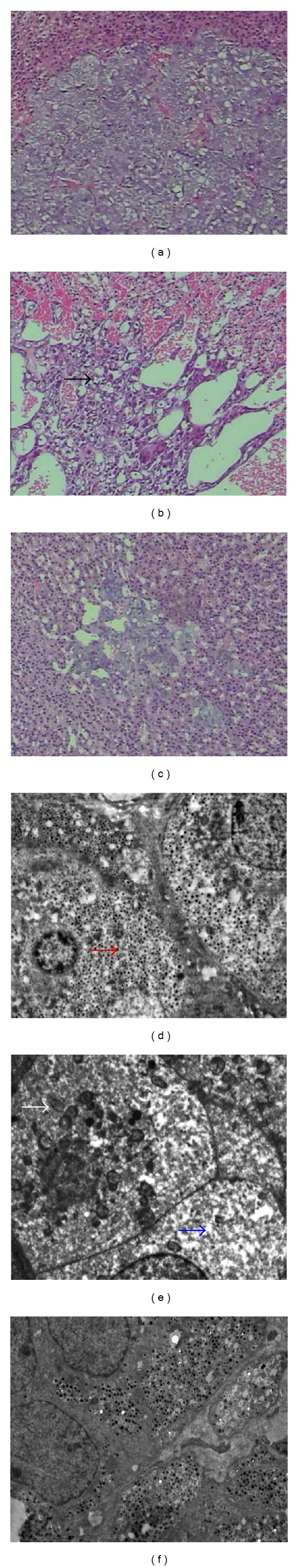
The pathological changes of rat adrenal medulla in response to induced asthma and KTR treatment. Representative micrographs (HE staining, 100x) of adrenal medulla in control rats (a), asthmatic rats (b), and KTR-treated asthmatic rats (c). Representative electron micrographs (10000x) of adrenal medulla in control rats (d), asthmatic rats (e), and KTR-treated asthmatic rats (f). The arrows show vacuolar degeneration (black arrow), chromaffin granules (red arrow), swollen cytoplasm, and mitochondria (blue and white arrows), respectively.

Compared with the control rats, asthmatic rats presented with vacuolar degeneration, tissue edema, and increased lipid in AMCC, as observed under light microscope. However, these lesions were reversed by KTR treatment (Figures 3(a)–3(c)).

Electron microscopy revealed a regular morphology of AMCC in control rats, which contained a clear structure of mitochondria and abundant chromaffin granules. Lesions were evident in asthmatic rats, including swollen cytoplasm and mitochondria, and decreased chromaffin granules. These pathological changes were relieved in the KTR-treated rats (Figures 3(d)–3(f)).

### 3.4. Changes in EPI, Corticosterone, and NGF Levels

**Figure 4:**
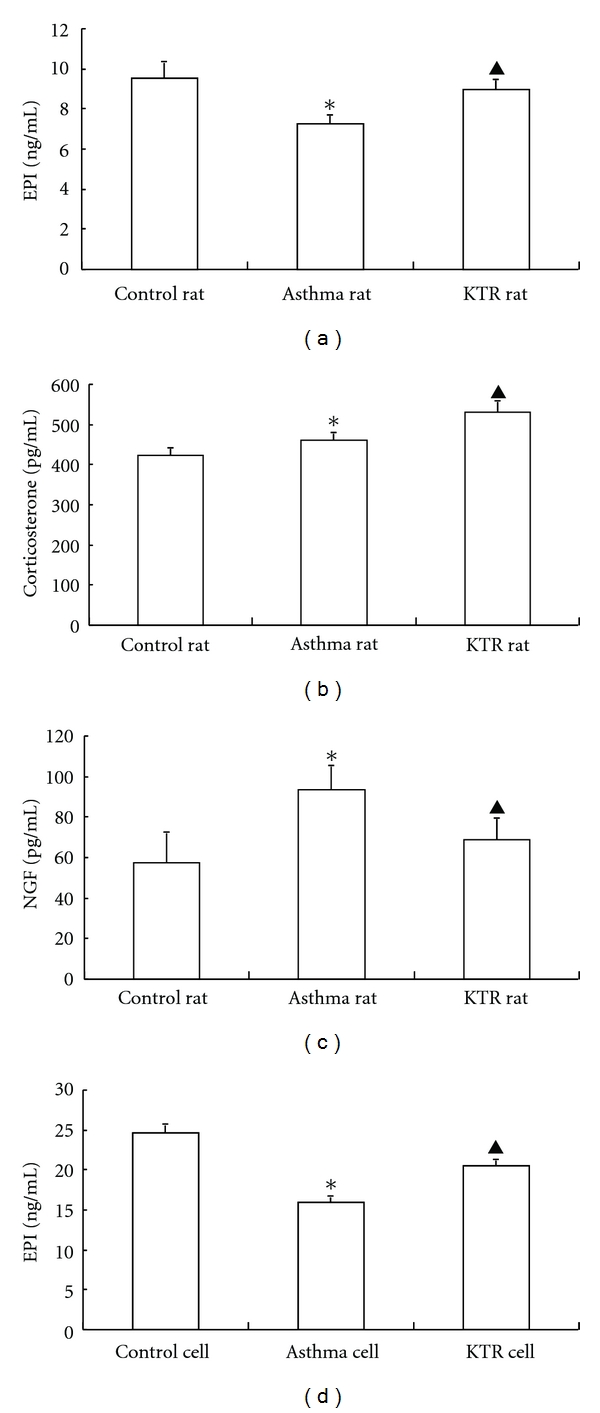
Changes in levels of EPI, corticosterone, and NGF in rats in response to induced asthma and KTR treatment. The levels of EPI, corticosterone, and NGF in rats (*in vivo*: (a), (b), and (c), resp.) and the level of EPI in cultured AMCC supernatant (*in vitro*: (d)) were detected by ELISA. The values are presented as mean ± SD (*in vivo*: *n* = 8, *in vitro*: *n* = 3). *indicates significant difference compared to the control group (*P* < 0.05); ^▲^indicates significant difference between KTR-treatment and asthma groups (*P* < 0.05).

EPI levels in asthmatic rats were lower than in control rats (*P* < 0.05), as evidenced by ELISA. Compared with asthmatic rats, the EPI levels increased significantly in KTR-treated rats (*P* < 0.05) (Figure 4(a)). Corticosterone levels were increased in asthmatic rats, as compared to that in control rats (*P* < 0.05), and corticosterone levels were further increased in asthmatic rats treated with KTR (*P* < 0.05) (Figure 4(b)). NGF levels were also increased in asthmatic rats, as compared to control rats (*P* < 0.05), but this response was attenuated in asthmatic rats by KTR treatment (*P* < 0.05) (Figure 4(c)).

### 3.5. Changes in PNMT and Peripherin Protein Expressions in Adrenal Medulla

**Figure 5:**
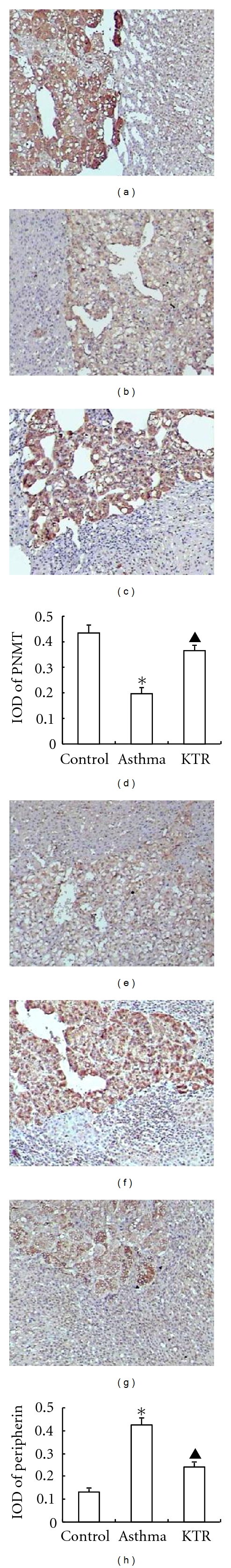
Changes in PNMT and peripherin protein expression in adrenal medulla of rats in response to induced asthma and KTR treatment. The expressions of PNMT and peripherin in the adrenal medulla of rats were detected by immunohistochemistry. Representative images of immunostaining (DAB, 100x) for PNMT are presented for control rats (a), asthmatic rats (b), and KTR-treated asthmatic rats (c). Representative images of immunostaining (DAB, 100x) for peripherin are presented for control rats (e), asthmatic rats (f), and KTR-treated asthmatic rats (g). Bar graphs on the right panels represent the average optical density of PNMT (d) and peripherin (h). The values are presented as means ± SD (*n* = 8). *indicates significant difference compared to the control group (*P* < 0.05); ^▲^indicates significant difference between KTR-treatment and asthma groups (*P* < 0.05).

Under normal physiologic conditions, PNMT is primarily expressed in chromaffin cells and absent in neurons. We found that the immunostaining of PNMT was distributed throughout cytoplasm. In control rats, the density of immunostaining was remarkably high in the adrenal medulla. Compared with the control rats, the expression of PNMT in asthmatic rats was decreased (*P* < 0.05), while a further enhanced expression was found in asthmatic rats treated with KTR (*P* < 0.05) (Figures 5(a)–5(d)).

Peripherin is rarely expressed in chromaffin cells and has high expression in neurons. We found that peripherin immunostaining was mainly localized in the cytoplasm. Compared with control rats, the density of immunostaining in asthmatic rats was higher than that in control rats (*P* < 0.05). However, KTR treatment inhibited the expression of peripherin in asthmatic rats (*P* < 0.05) (Figures 5(e)–5(h)).

### 3.6. Changes in Peripherin Gene and Protein Expressions in the Adrenal Medulla

**Figure 6:**
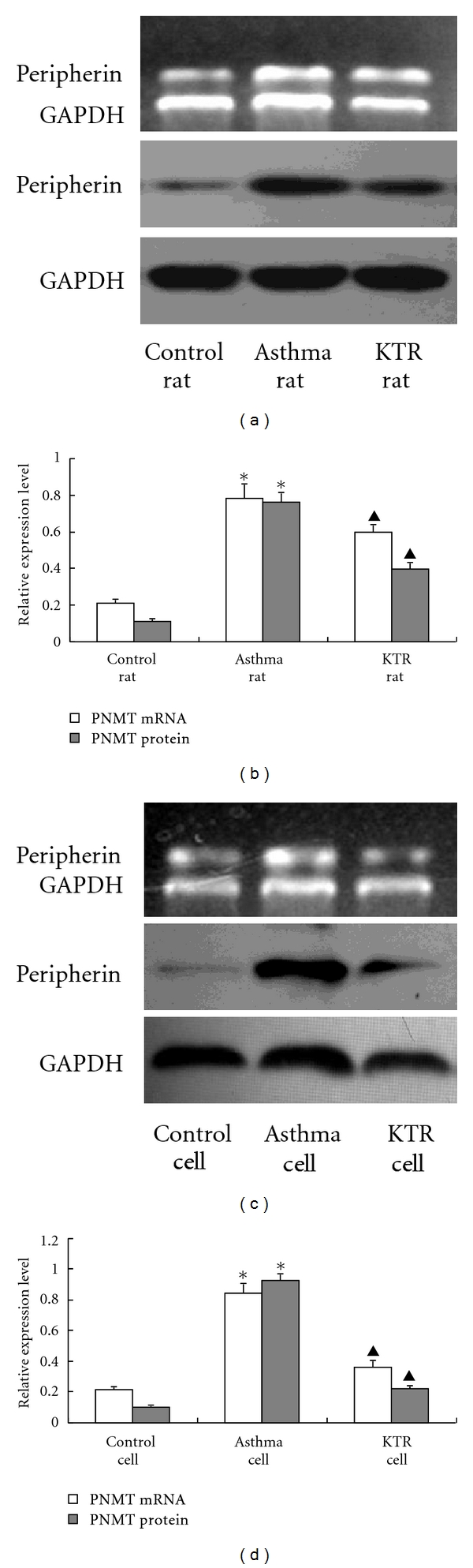
Changes in peripherin gene and proteins expressions in rat adrenal medulla tissue and AMCC in response to induced asthma and KTR treatment. Total RNA and cellular protein were isolated from adrenal medulla tissue in rats (*in vivo*: (a) and (b)) and AMCC (*in vitro*: (c) and (d)), and the expressions of peripherin (mRNA and protein) in each group were detected by RT-PCR and western blot. Results were normalized to GAPDH, which was used as an internal control, and the bar graphs on the right show the relative densitometry results. The values are expressed means ± SD (*in vivo*: *n* = 8, *in vitro*: *n* = 3). GAPDH was used as an internal control. *indicates significant difference compared to the control group (*P* < 0.05); ^▲^indicates significant difference between KTR-treatment and asthma groups (*P* < 0.05).

Compared with control rats, asthmatic rats had significantly increased amounts of peripherin mRNA and protein expression (*P* < 0.05), as evidenced by RT-PCR and western blot, respectively. Compared with the asthmatic rats, peripherin mRNA and protein expression was inhibited by KTR treatment (*P* < 0.05) (Figures 6(a) and 6(b)).

### 3.7. AMCC Structural Alterations *In Vitro*

**Figure 7:**
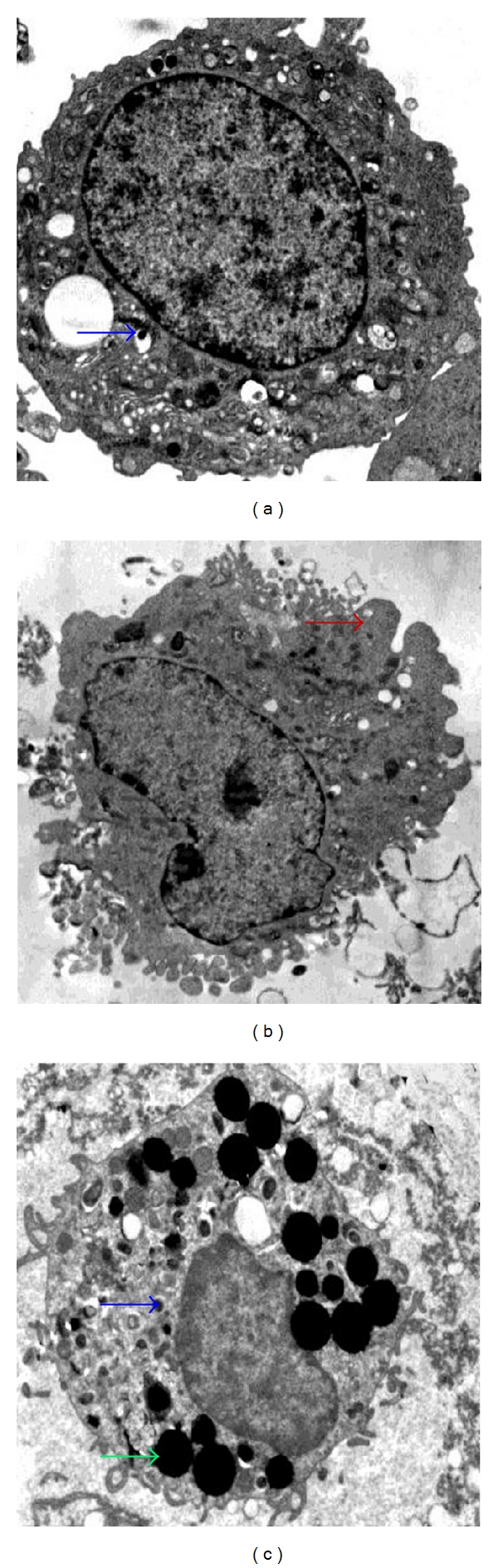
Pathological changes of AMCC *in vitro*. Representative electron micrographs (20000x) of AMCC treated with control serum (a), asthma serum (b), and KTR serum (c). The arrows show chromaffin granules (blue arrow), neurite-like process (red arrow), and lysosome (green arrow), respectively.

Electron microscopy indicated that cultured chromaffin cells treated with control serum* in vitro* were of regular shape and contained abundant chromaffin granules. A decrease in the amount of chromaffin granules and neurite-like processes and in swollen cytoplasm and mitochondria appeared in cells upon treatment with asthmatic serum, as compared with control cells. These pathological changes were alleviated with treatment with KTR serum, and there were numerous lysosomes present in the cytoplasm (Figure 7).

### 3.8. EPI Levels in Supernatants of Cultured AMCC

Compared with cells treated with control serum, EPI levels were sharply decreased in the supernatant of cells treated with asthmatic serum (*P* < 0.05), as evidenced by ELISA. This was reversed by KTR serum treatment (*P* < 0.05) (Figure 4(d)).

### 3.9. Peripherin Gene and Protein Expressions in Cultured AMCC

Compared with the control cells, AMCC treated with asthmatic serum showed a significant increase in peripherin mRNA and protein expressions (*P* < 0.05), as evidenced by RT-PCR and western blot, respectively. This increase was inhibited by KTR serum (*P* < 0.05) (Figures 6(c) and 6(d)).

## 4. Discussion

Kidney-tonifying drugs are often used to treat asthma in TCM. Previous reports have suggested that the effects of these drugs are mediated by their ability to upregulate plasma cortisol production by stimulating the adrenal cortex [3]. The KTR formula contained Rehmannia Glutinosa and medicinal Indian mulberry root, which have each been shown to improve adrenal cortex function and to increase glucocorticoid receptor expression, thereby relieving asthma [16]. Furthermore, monkshood could act as a *β*_2_-adrenergic receptor agonist, which can relieve bronchoconstriction in asthma [17]. As well as glucocorticoids, EPI and the other *β*_2_-adrenergic receptor agonists play an important role in treating asthma. However, it is not known whether kidney-tonifying drugs affect the secretion of EPI. Asthmatic patients have been clinically shown to have decreased levels of endogenous EPI, which may be the important reason for supplementing exogenous EPI in relieving asthma [8, 9]. In the present study, EPI concentrations were found to be reduced in asthmatic rats, and KTR administration attenuated this decrease. Furthermore, EPI levels were further found to be correlated with the degree of bronchial hyperreactivity and lung lesions in asthmatic rats. These results indicated that the mechanism underlying KTR effects in treating asthma is mediated by regulating EPI concentration.

EPI is a *β*_2_-adrenergic receptor agonist that is mainly secreted from the adrenal medulla [6, 7]. The adrenal gland consists of cortex and medulla, the latter of which is an endocrine organ that originates from the neuroectoderm. During early embryonic periods, the development of adrenal medulla is parallel with that of the sympathetic nerves. In later stages the adrenal medulla invades the adrenocortical rudimentum, at which time the adrenal medulla gains its characteristic endocrine secretion properties [18]. The homology between the adrenal medulla and sympathetic nerves underlies the ability of AMCC to transform to sympathetic neurons, and this is induced by the activities of nerve growth factor (NGF) [18–20]. It is well known that sympathetic neurons synthesize and secrete noradrenaline and dopamine, but not adrenaline. As a result, the transition to neurons inhibits the secretion of EPI in AMCC.

NGF is the most important neurotrophic factor involved in neuronal growth, survival, and differentiation. NGF expression and activity is enhanced in asthma [21], and NGF levels are consistent with the degree of bronchial hyperreactivity [22]. In our previous studies, we observed a trend toward transformation from the AMCC phenotype to that of neurons in asthmatic rats. This was accompanied by a decrease in EPI and elevation in NGF, and anti-NGF inhibited this transformation. These data indicated that the decrease in EPI results from a phenotype transformation of AMCC to neuron, which is promoted by elevated NGF in asthma [10–12, 23]. In the present study, decreased EPI and elevated NGF were also found in asthmatic rats, and there was a decrease in the density of chromaffin granules, and swollen cytoplasm and mitochondria also appeared in AMCC of asthmatic rats. KTR treatment increased EPI levels, decreased NGF levels, and alleviated the ultrastructural lesions in AMCC.

During the development and regeneration of nerve cells, peripherin (a type III intermediate filament) plays an important role in cellular architecture through regulating axon formation, thus giving nerve cells their unique morphology [24]. The importance of peripherin in this development has been highlighted by a research study that observed that depletion by peripherin-siRNA inhibited the initiation, extension, and maintenance of neuritis [24]. Only a subpopulation of AMCC are reactive to peripherin and this is supported by the fact that AMCC expressing high peripherin levels have a distinct shape that is similar to that of nerve cells [25]. AMCC have also been implicated in active downregulation of intermediate filament proteins during embryonic development [18]. However, Nie found that the expression of peripherin was increased in the adrenal medulla of asthmatic rats, and in this process decreased chromaffin granules and neurite-like processes appeared in AMCC of asthmatic rats [23]. These data suggested that increased peripherin expression may be a hallmark of AMCC transformation in asthma. In the present study, enhanced expression of peripherin was observed in the adrenal medulla of asthmatic rats, which was normalized by KTR treatment. The results suggest that the beneficial effects of KTR in treating asthma are mediated by downregulation of peripherin expression in AMCCs, thus inhibiting the transformation of AMCCs to neurons.

PNMT, the rate-limiting enzyme that catalyzes nor-EPI to EPI, is mainly expressed in adrenergic chromaffin cells, rather than in nerve cells. Decreases in PNMT mediate the transition from AMCCs to neurons. Glucocorticoids are known to promote the expression of PNMT in the adrenal medulla [26–28]. In our recent study, we found that the PNMT expression was reduced in the adrenal medulla of asthmatic rats, and NGF administration intensified this effect [10]. High concentrations of glucocorticoids in the adrenal medulla have been shown to prevent fiber outgrowth from medullary chromaffin cells *in vivo* and inhibit the transformation of AMCC into neurons [15, 29, 30]. These results further support the importance of NGF and glucocorticoids in AMCC fate [15, 31].

In the current study, PNMT expression was found to be decreased in asthmatic rats, consistent with decreased EPI and increased NGF levels, and KTR treatment inhibited these changes. Despite increased corticosterone levels in asthmatic rats, there remained a trend for NGF-induced transformation of AMCC. The further increase in corticosterone in asthmatic rats treated by KTR may act as a compensatory mechanism, as supplementation with exogenous glucocorticoids is a highly effective treatment in asthma patients. To some extent, these data suggest that the effect of KTR in treating asthma is dependent on maintaining the phenotype of AMCC, which may be ascribed to correcting the imbalance of NGF and glucocorticoids; as a result, EPI concentrations would be expected to be increased, thus relieving bronchospasms.

To test this hypothesis, we cultured AMCC *in vitro* and treated them with sera from control, asthmatic, or KTR-treated rats. The results demonstrated that treatment with sera from asthmatic rats caused a decrease in the density of chromaffin granules and development of a synapse-like cellular structure. In addition, asthmatic sera also caused a concurrent decrease in EPI and increase in peripherin. These changes were attenuated in AMCCs treated with the sera from KTR-treated rats.

In summary, it can be concluded that administration of KTR to asthmatic rats leads to upregulation of EPI and prevention of AMCC transformation to neurons, thus allowing for normal endocrine function.
